# Elective Splenectomy Combined with Modified Hassab's or Sugiura Procedure for Portal Hypertension in Decompensated Cirrhosis

**DOI:** 10.1155/2019/1208614

**Published:** 2019-04-28

**Authors:** Ya-wu Zhang, Feng-xian Wei, Zhen-gang Wei, Gen-nian Wang, Man-cai Wang, You-cheng Zhang

**Affiliations:** ^1^Department of General Surgery, Lanzhou University Second Hospital, Lanzhou 730030, China; ^2^Hepato-Biliary-Pancreatic Institute, Lanzhou University Second Hospital, Lanzhou 730030, China; ^3^Lanzhou University Second Clinical Medical College, Lanzhou University, Lanzhou 730030, China

## Abstract

**Objective:**

Portal hypertension is a major complication of decompensated cirrhosis. In China, modified Hassab's and Sugiura procedure are the two major methods of nonshunting surgery. This study aims to compare the efficacy and safety of the two procedures for portal hypertension.

**Method:**

Between January 1994 and December 2009, 172 elective patients diagnosed with decompensated cirrhosis with significant hypersplenism adopted elective splenectomy for hypersplenism, and also modified Hassab's (*n *= 91) or Sugiura (*n *= 81) procedure was additionally performed to reduce the risk of variceal bleeding. Postoperative mortality and morbidity data were collected, and a retrospectively comparative analysis was conducted.

**Results:**

All of the patients were treated successfully without death during operation, and no variceal bleeding occurred during hospitalization. There were 4 (4.4%) deaths in Hassab's group and 3 (3.7%) deaths in Sugiura group postoperatively (P > 0.05). During follow-up, the survival rate was 90.2%, 82.42%, and 71.43% in Hassab's group and 96.29%, 81.48%, and 75.31% in Sugiura group in 1, 3, and 5 years (P > 0.05). There were 22/71 and 12/63 patients in each groups who suffered no deadly variceal bleeding (P = 0.11). Bleeding related death and no bleeding related death occurred in 7/23 and 3/13 patients in each group (P = 0.26 and 0.14, respectively).

**Conclusion:**

Elective splenectomy combined with modified Sugiura procedure seemed to be associated with a reduced trend of no deadly variceal bleeding compared with Hassab's procedure. As statistical significance was not found, further large scale and prospective study was warranted.

## 1. Introduction

Portal hypertension is mainly caused by liver cirrhosis, which usually represents the end-stage of chronic hepatitis due to virus infection, alcohol abuse, fatty liver disease, and other kinds of diseases [1]. The prevalence of liver cirrhosis is reported to be increasing that it is the 23rd cause of deaths in 2010 and 12th cause of deaths in 2011, whereas the related disease burden is underestimated [1, 2]. Its consequences include gastroesophageal variceal hemorrhage, ascites, spontaneous bacterial peritonitis, hepatorenal syndrome, hepatopulmonary/portopulmonary syndromes, and hepatic encephalopathy. To be the first cause of death in decompensated cirrhosis patients, variceal hemorrhage always occurred in patients with moderate-to-severe red color sign in esophagus, which accounted for approximately 12% in total patients, and 60% of them would experience a recurrent hemorrhage [3, 4]; in this phase, the mortality rate would be as high as 57% in a year [5]. Three main challenges exist for the management of portal hypertension related esophageal varices in clinical practice: the prevention of first episode of hemorrhage, the treatment of acute bleeding episodes, and the prevention of recurrent variceal hemorrhage [3].

Comprehensive treatment including drug therapy [6], endoscopic techniques (endoscopic injection sclerotherapy and endoscopic variceal ligation), interventional radiology (transjugular intrahepatic portosystemic stent-shunt [TIPS], percutaneous transhepatic obliteration, transileocolic vein obliteration, and partial splenic artery embolization), surgery, liver transplantation, and a combination of the former methods have been developed during the past years [7, 8]. Among them, both shunting and nonshunting surgery can achieve stable control for both first and recurrent variceal hemorrhage and also can be applied when other new developed and less invasive techniques failed. It was reported that nonshunting surgery had a higher risk of rebleeding than shunting surgery [9, 10], with no significant difference of mortality. Besides, nonshunting surgery is much more simple in technique and is associated with a lower risk of encephalopathy [11].

As interventional radiology such as TIPS was widespread, while it did not contribute to many benefits on hypersplenism besides variceal bleeding treatment. Particularly, hypersplenism together with collateral circulation is very common in China; elective splenectomy combined with modified Hassab's or Sugiura procedure is thus very popular for portal hypertension in decompensated cirrhosis patients. Meanwhile, previous study indicated surgery a new role for cirrhotic patients as it treated several pathological processes caused by liver cirrhosis [12]. Together with the increasing burden of liver cirrhosis, it is necessary to reevaluate the clinical outcomes of the two procedures. In this study, we retrospectively compared perioperative and postoperative data of elective splenectomy combined with modified Hassab's procedure and Sugiura procedure in both bleeding related mortality and nonbleeding related mortality as well as morbidity to clarify their effectiveness in the management of portal hypertension.

## 2. Patients and Methods

### 2.1. Patients and Grouping

From January 1994 to December 2009 in Lanzhou University Second Hospital, 172 patients diagnosed with portal hypertension with a high risk of variceal hemorrhage who underwent nonshunting surgery were retrospectively analyzed. Inclusion criteria included (1) age < 60 years, (2) severe splenomegaly and hypersplenism that required splenectomy, (3) prothrombin activity > 40%, (4) Child-Pugh classification of B and C or recovery to A after proper treatment preoperatively, and (5) risk of first variceal bleeding (moderate-to-severe red color sign in esophagus by endoscopy). Exclusion criteria included (1) portal hypertension without hypersplenism, (2) emergency patients without sufficiently preoperative evaluation and treatment, (3) bleeding patients failed to be controlled by other less invasive technique, especially for endoscopic injection sclerotherapy failure due to its certainly negative effects on anastomosis, (3) uncontrolled and serious white blood cell, hemoglobin, and platelet decrease after transfusion or other drugs, (4) hepatocellular carcinoma, and (5) heart, liver, and kidney dysfunction not able to tolerate general anesthesia and surgery injury. According to inclusion and exclusion criteria, 91 patients who adopted modified Hassab's procedure were included into Hassab's group, and 81 patients who adopted modified Sugiura procedure were in the Sugiura group. Informed consent was required before surgery. The study was legally approved by our Institutional Ethics Committee. The rights and specific information of the all the participants were protected in the process of both data collection and analysis.

### 2.2. Surgical Procedures

Firstly, current surgery is based on the necessity of hypersplenism treatment and secondly for lowering the risk of deadly variceal bleeding. The indication of splenectomy for hypersplenism was mainly based on the platelet count. White blood cells (WBC) and platelet (PLT) count can be easily influenced in hypersplenism, and among them platelet count is earlier and more sensitive than white cells in hypersplenism patients with different severity. Also, most patients with low white blood cells can be commonly and effectively controlled by drugs in clinic. So, current study restricted the indication of splenectomy to patients with PLT count <80 × 10^12^/L and ineffective leukopenia by drugs.

General preoperative preparation included prophylactic antibiotics, cleaning enema, gastrointestinal decompression, and infusion. Both of the procedures were completed by three different hepatobiliary surgeons (Prof. YC Zhang, Prof. YW Zhang, and Prof. ZG Wei) with other assistants in the team; all the surgeons had more than 5-year experience of more than 20 cases of such operations. The distribution of the two procedures was same over time in our center.

Modified Hassab's procedure mainly focused on selective vascular devascularization [13]. Extensive devascularization included pericardial and upper stomach vascular type (ablations of the left gastroepiploic vein, short gastric vein, vena gastric posterior, left inferior phrenic vein, left gastric vein, and high branches and probable ectopic branches of gastric coronary vein). And after devascularization, serosa wall of the stomach was sutured. Modified Sugiura procedure included transabdominal gastroesophageal devascularization and additional esophageal transection/anastomosis, which theoretically ensured a more comprehensive devascularization of transesophageal and submucosal vessels [14]. Similar to the above extensive vascular devascularization, the distal esophagus 4-6cm above the gastric cardia was additionally transected and anastomed with a stapler.

### 2.3. Data Collection and Follow-Up

Baseline characteristics of the patients were retrospectively collected, including average age, sex, and etiology, the history of upper gastrointestinal bleeding, Child-Pugh classification, white blood cell, platelet, direct bilirubin, indirect bilirubin, albumin, prothrombin activity, fibrinogen, free portal pressure, and operation time. Outcome measurements included perioperative bleeding, mortality and causes, postoperative rebleeding, survival, mortality and causes, and morbidity and diagnosis. All perioperative outcomes were measured during hospital stay, and postoperative outcomes were measured after discharge and follow-up for 5 years ranging between 1, 3, and 5 years or until death through telephone or outpatient department visit.

### 2.4. Statistical Analysis

Data analysis was carried out by using SPSS soft (Version 12.0, SPSS Inc., Chicago, IL, USA) for Windows. For patients lost to follow-up, statistical analysis excluded the data in specific outcome measures. Continuous variables were expressed as mean ±standard deviation and compared using an nonparametric Manne-Whitney* U* test. Dichotomous variables were presented as case and percentage (%), and the significance was obtained by *χ*^2^ test.* P*< 0.05 was considered statistically significant.

## 3. Results

### 3.1. Patients Characteristics

From January 1994 to December 2009, 172 patients underwent nonshunting surgery. There were 63 male patients and 28 female patients in Hassab's group, and the average age was 42.3 ± 13.24 years, ranging from 17 to 64 years. There were 60 male patients and 21 female patients in Sugiura group, and the average age was 43.1 ± 15.21 years, ranging from 21 to 68 years. The etiologies were post-hepatitis A cirrhosis (6/5), post-hepatitis B cirrhosis (67/70), post-hepatitis C cirrhosis (15/5), alcoholic cirrhosis (2/1), and autoimmune cirrhosis (1/1) in the two groups, respectively. Preoperative history of acute upper gastrointestinal bleeding occurred in 88 patients in Hassab's group and 68 patients in Sugiura group, and all of the 156 patients were diagnosed with splenomegaly and hypersplenism. Preoperative functional hepatic reserve was classified according to the Child-Pugh classification, and the cases in the two groups of Grade A, Grade B, and Grade C were 6/9, 67/58, and 14/11, respectively. The preoperative levels of white blood cell, platelet, direct bilirubin, indirect bilirubin, albumin, prothrombin activity, and fibrinogen were listed in Table 1, and no significant difference was found between the groups. Average free portal pressure was 44 ± 3.5 and 43 ± 3.8 cm H_2_O. Modified Sugiura procedure was associated with significantly longer operation time than that of modified Hassab's procedure (166.2 ± 48.1 vs 210.5 ± 62.3 min, P < 0.05). No statistically significant difference was found to be located in baseline data between the groups as shown in Table 1.

### 3.2. Perioperative Bleeding and Mortality

All of the patients were treated successfully without death occurring during operation process, and no incidence of variceal bleeding occurred during hospitalization. In Hassab's group, there were 4 deaths including 2 cases of massive ascites with renal failure, 1 case of stress ulcer bleeding, and 1 case of liver function failure, with an overall mortality of 4.4% (4/91). In Sugiura group, there were 3 deaths including 1 case of massive ascites with renal failure, 1 case of liver function failure, and 1 case of acute respiratory distress syndrome, with an overall mortality of 3.7% (3/81). No significant difference was found between the groups (Table 2).

### 3.3. Mortality and Variceal Bleeding Related Mortality during Follow-Up

A total of 87 patients in Hassab's group and 78 patients in Sugiura group were finally discharged after improvement from hospital. The data of 16 patients and 15 patients in the two groups were absent after 1-year follow-up, who were regarded as lost to follow-up; thus the follow-up rate was 81.6% and 80.8%, respectively. The 1-, 3-, and 5-year survival rates were 90.2%, 82.42%, and 71.43% in Hassab's group and 96.29%, 81.48%, and 75.31% in Sugiura group, respectively. No significant difference was shown in follow-up rate and survival rate. Among them, 7 (7.69%) and 3 (3.70%) in each group suffered deadly variceal bleeding, and the difference also failed to be statistically significant. Besides, 23 and 13 patients were dead because of no variceal bleeding related causes including liver cancer, hepatic failure, and renal failure, as shown in Table 3.

### 3.4. Morbidity and Variceal Bleeding Related Morbidity during Follow-Up

For bleeding prevention, there were 22 (31%) patients in Hassab's group and 12 (19%) patients in Sugiura group who suffered postoperative variceal bleeding. Although higher incidence seemed to be associated with Hassab's procedure, the difference did not reach statistical significance (P > 0.05). Other morbidities included hepatic encephalopathy (12/7), portal vein thrombosis (3/2), ascites (18/9), pleural effusion (5/3), anastomotic stricture (0/3), and gastric retention (0/4). No significant difference was found in the aspects of both overall and specific morbidities, as shown in Table 4.

## 4. Discussion

Portal hypertension is mainly caused by liver cirrhosis which is reported to be with a prevalence of 0.15% in the United States and European countries, and much more patients mainly located in Asia and Africa [15]. Collateral circulation, splenomegaly, and hypersplenism are three major syndromes, which always lead to medical emergency of variceal bleeding, hematologic syndrome of thrombocytopenia, and/or leukopenia [16]. Liver transplantation is considered to be the most effective method to treat portal hypertension in decompensated period [17]. However, organ shortage and high medical cost appear to be the major problem in clinical transplantation currently, especially in China.

In the management of portal hypertension, emergency procedure for life-threatening variceal bleeding must to be done firstly, and an appropriate procedure is supposed to have a low mortality, a low rate of rebleeding rate, a low incidence of hepatic coma, and less decompensation of hepatic function. Currently, drugs, endoscopic techniques, and interventional radiology with reliable efficacy are firstly recommended [7, 18]. Emergency surgery may be required only for patients when endoscopic and pharmacologic therapies are unsuccessful and TIPS placement is contraindicated, unavailable, or unsuccessful [14].

Meanwhile, elective surgery is mainly recommended to patients who are not scheduled for liver transplantation and with certainly severe splenomegaly and hypersplenism requiring splenectomy [16]. Actually, there is no need to perform a portal hypertension surgery (shunting or nonshunting surgery) aiming at only preventing or treating variceal bleeding nowadays. Based on the requirement of splenectomy, reports stated that surgeons tend to choose their favorite portal hypertension surgery as optimal procedures mainly based on patient's conditions and operator's skill [19]. Nonshunting surgery is certainly easier in technique than shunting surgery. Currently, hepatitis B virus is the most common cause of portal hypertension and liver cirrhosis in China and further accompanies hypersplenism commonly; thus splenectomy combined with nonshunting surgery is the most popular choice [11, 20].

Current study retrospectively compared two kinds of nonshunting surgery named modified Hassab's and Sugiura operation focused on bleeding, mortality, and morbidity outcomes for elective patients. The average operation time was longer in the Sugiura group than that in Hassab's group. However, our data showed that bleeding related outcome measurements are equivalent between them, although there seemed to be a trend without statistical significance of a higher incidence of no deadly variceal bleeding after Hassab's procedure than Sugiura procedure. Modified Sugiura procedure considering existence of patent perforating veins and periesophageal collaterals is the main reason of esophageal variceal recurrence and rebleeding [21, 22]; thus additional esophageal transection and anastomosis are performed in order to achieve a relative more thorough devascularization. Also, modified Sugiura procedure is relatively complex and may suffer high injury stress than Hassab's procedure. Although a higher incidence of anastomotic stricture occurred in the Sugiura group, no statistical difference was found in all of the no variceal bleeding related mortality and morbidity. Compared with other studies [19, 20], the mortality in our study was lower; it may be because our study excluded emergency patients due to variceal bleeding. In this study, treatment of hypersplenism is the primary purpose and concern, and prevention of the first or recurrent variceal bleeding is additionally performed. About 13% of patients in Hassab's group and 16% of patients in Sugiura group did not have a history of bleeding, and nearly all the patients did not have an average level of platelet within normal limits. Besides, our patients seemed to have a higher proportion of A and B patients under Child-Pugh classification.

### 4.1. Implications

Improved vasoconstrictor such as octreotide, somatostatin, and vapreotide and modified surgery with reduced invasion (not only modified surgery procedure, but also laparoscopic instrument application) decreased the incidence of perioperative mortality and morbidity for portal hypertension patients [23]. During the past years, widespread application of other mini-invasive endoscopic and radiology techniques had obviously decreased the dependence of surgery for life-threatening variceal bleeding [7]; endoscopic techniques together with pharmacotherapy remained the first-line treatment with >85% bleeding control [14]. Thus, it seems that a combination of other techniques may achieve equivalent overall treatment effects [16, 18, 21]. Actually, for portal hypertension patients suffering hypersplenism complicated by high risks of gastroesophageal varices as well as liver dysfunction, they are difficult to control without elective splenectomy [14]. Under this situation, additional nonshunting surgery was considered by our center to ensure an extensive devascularization including pericardial and upper stomach vascular types, which could be hardly completed by other techniques. Because of the development of less invasion concept, modified laparoscopic nonshunting surgery has been introducing and playing new roles in the management of portal hypertension [24, 25]. Although invasion is less, the specific surgery scope and method do not have essential difference between open and laparoscopic surgery other than surgery-related complications [25]. Dr. Wang et al. retrospectively investigated the efficacy of the modified Sugiura procedure and Hassab procedure for treatment of rebleeding due to portal hypertension after endoscopic variceal ligation and found the modified Sugiura procedure is more effective than the Hassab procedure for the treatment of rebleeding after endoscopic variceal ligation [26]. Current study compared two open nonshunting procedures and showed a potential trend of decreased no deadly variceal bleeding in modified Sugiura procedure, which would be helpful in emphasizing the necessity of additional esophageal transection and for further research although sufficient evidence is absent.

### 4.2. Limitations

Emergency patients failed to be controlled by endoscopic technique and pharmacotherapy were excluded; the limitations from study design and participant inclusion may induce unavoidably little bias. Also, preoperative evaluation of liver function was mainly based on Child-Pugh classification, which is easy to realize but is relatively inaccurate compared with new developed indocyanine green retention test (ICG-R15) [27]. Although controversial results existed in aspects of the portal vein pressure changes, compared with shunting surgery nonshunting aiming at lowering the risk of life-threatening bleeding would not significantly reduce the portal vein pressure [20]. But a series of small-scale observational studies found that, after surgery, portal venous flow was decreased while hepatic artery flow was increased in compensation, and this phenomenon is so interesting that it could to some extent increase the functional hepatic reserve and promote hepatitis recovery with significantly improved parameters and ICG-R15 level [16, 28, 29]. Meanwhile, more and more studies showed that splenectomy would induce a progressive improvement of liver function parameters and may improve liver fibrosis and cause beneficial immunological changes in cirrhotic patients with hepatitis [16, 30]. Although current analysis did not involve any issue of mentioned aspects, our current study is during follow-up periods aiming at investigating the effects as well as liver regeneration, especially for most of current patients without chance to receive liver transplantation limited by medical and financial reasons.

## 5. Conclusions

Elective splenectomy combined with modified Sugiura procedure seemed to be associated with a reduced trend of no deadly variceal bleeding compared with Hassab's procedure. Although statistical significance was not found, large scale and prospective study was warranted.

## Figures and Tables

**Table 1:** Baseline characteristics of the included patients.

Group	Hassab's group (*n *= 91)	Sugiura group (*n *= 81)	*P *value
Sex (*n, %*)			
Male	63 (69.23%)	60 (70.07%)	> 0.05
Female	28 (30.77%)	21 (25.93%)	> 0.05
Age (yrs)	42.3 ± 13.24	43.1 ± 15.21	> 0.05
Etiology (*n, %*)			
Post-hepatitis A cirrhosis	6 (6.59%)	5 (6.17%)	> 0.05
Post-hepatitis B cirrhosis	67 (73.63%)	70 (86.42%)	> 0.05
Post-hepatitis C cirrhosis	15 (16.48%)	5 (6.17%)	> 0.05
Alcohol	3 (3.30%)	2 (2.47%)	> 0.05
Autoimmune cirrhosis	1 (1.09%)	1 (1.23%)	> 0.05
The history of upper gastrointestinal bleeding (*n, %*)	79 (86.81%)	68 (83.95%)	> 0.05
Child-Pugh classification (*n, %*)			
A	6 (6.59%)	9 (11.11%)	> 0.05
B	67 (73.63%)	58 (71.60%)	> 0.05
C	14 (15.38%)	11 (13.58%)	> 0.05
White blood cell (×10^9^/L)	3.8 ± 2.2	5.1 ± 3.1	> 0.05
Platelet (×10^9^/L)	47 ± 18	45 ± 19	> 0.05
Direct bilirubin (umol/L)	6.7 ± 4.5	6.9 ± 4.8	> 0.05
Indirect bilirubin (umol/L)	26.6 ± 4.8	28.0 ± 5.3	> 0.05
Albumin (g/L)	36.2 ± 9.0	35.3 ± 10.2	> 0.05
Prothrombin activity (%)	69.7 ± 17.5	68.6 ± 16.1	> 0.05
Fibrinogen (g/L)	1.82 ± 0.70	1.90 ± 0.74	> 0.05
Free portal pressure (cm H_2_O)	44 ± 3.5	43 ± 3.8	> 0.05
Operation time (min)	166.2 ± 48.1	210.5 ± 62.3	< 0.05

**Table 2:** Perioperative incidence and causes of mortality.

Group	Hassab's group	Sugiura group	*P *value
	(*n *= 91)	(*n *= 81)	
Post-operative mortality (*n, %*)	4 (4.4%)	3 (3.7%)	0.82
Causes of death (*n*)			
Massive ascites with renal failure	2	1	0.63
Stress ulcer bleeding	1	1	0.34
Liver function failure	1	0	0.29
Acute respiratory distress syndrome	0	1	0.29

**Table 3:** Incidence of mortality during follow-up.

Group	Hassab's group	Sugiura group	*P *value
	(*n *= 91)	(*n *= 81)	
Survival rate			
1 year	82 (90.20%)	78 (96.29%)	0.11
3 years	75 (82.42%)	66 (81.48%)	0.87
5 years	65 (71.43%)	61 (75.31%)	0.57
Causes of death			
Hepatic failure	13 (14.29%)	9 (11.11%)	0.53
Liver cancer	8 (8.79%)	2 (2.47%)	0.08
Renal failure	2 (2.20%)	2 (2.47%)	0.91
Upper gastrointestinal bleeding	7 (7.69%)	3 (3.70%)	0.26
Mortality			
Variceal bleeding related	7 (7.69%)	3 (3.70%)	0.26
No bleeding related	23 (25.27%)	13 (16.05%)	0.14

**Table 4:** Incidence of morbidity during follow-up.

Group	Hassab's group	Sugiura group	*P* value
	(*n* = 71)	(*n* = 63)	
No deadly upper gastrointestinal bleeding (*n, %*)	22 (30.96%)	12 (19.05%)	0.11
Portal vein thrombosis (*n, %*)	3 (4.23%)	2 (3.17%)	0.75
Ascites (*n, %*)	13 (18.31%)	9 (14.29%)	0.53
Hepatic encephalopathy (*n, %*)	12 (16.90%)	7 (11.11%)	0.34
Pleural effusion (*n, %*)	5 (7.04%)	3 (4.76%)	0.58
Gastric retention (*n, %*)	1 (1.41%)	4 (6.35%)	0.13
Anastomotic stricture (*n, %*)	0	3 (4.76%)	0.06

## Data Availability

The analyzed data in the manuscript used to support the findings of this study are available from the corresponding author upon request.
